# Hybrid Clinical and Histopathological Pattern in Annular Lesions: An Overlap between Annular Elastolytic Giant Cell Granuloma and Granuloma Annulare?

**DOI:** 10.1155/2012/102915

**Published:** 2012-10-15

**Authors:** Felipe Ladeira de Oliveira, Luisa Kelmer Côrtes de Barros Silveira, Alice de Miranda Machado, José Augusto da Costa Nery

**Affiliations:** ^1^Fundação Oswaldo Cruz (FIOCRUZ), Avenida Brasil, 4365 Manguinhos, 21040-360 Rio de Janeiro, RJ, Brazil; ^2^Ambulatório Souza-Araújo, Leprosy Department, Fundação Oswaldo Cruz (FIOCRUZ), 21040-360 Rio de Janeiro, RJ, Brazil

## Abstract

Annular elastolytic giant cell granuloma (AEGCG) is a rare granulomatous skin disease of unclear pathogenesis which belongs to the group of disorders in the skin and elastic fibers with similar clinical features of granuloma annulare (GA). This case report is intended to describe a rare hybrid pattern in histopathology demonstrating coexistence of AEGCG and GA. An endocrine disease, such as diabetes mellitus (DM), could contribute to the coexistence of both lesions, and this possibility must be included in the medical investigation.

## 1. Introduction

Annular elastolytic giant cell granuloma (AEGCG) is a rare granulomatous skin disease of unclear pathogenesis whichbelongs to the group of disorders inthe skin andelastic fiberswith similarclinical featuresof granuloma annulare (GA) and necrobiosis lipoidica [1]. Lesions of AEGCG are mostly found in middle-aged white women, usually located on sun-exposed areas and rarely on covered areas [2]. Clinically, AEGCG presents as papules and annular plaques with erythematous bordersand withatrophichypopigmented center growingcentrifugally [3]. The chronic course of this disease is a typical feature as the variable response to existing treatments [4].

Therefore, this case report is intended to describe and discuss a case of AEGCG, irresponsive to the treatment, associated with diabetes and rare hybrid pattern in histopathology demonstrating coexistence of AEGCG and GA.

## 2. Case Presentation

**Figure 1:**
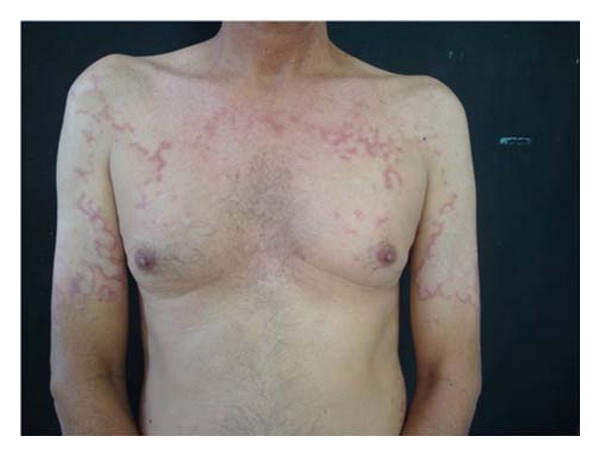
Atrophic plaques with slightly elevated border. Observe the annular pattern.

**Figure 2:**
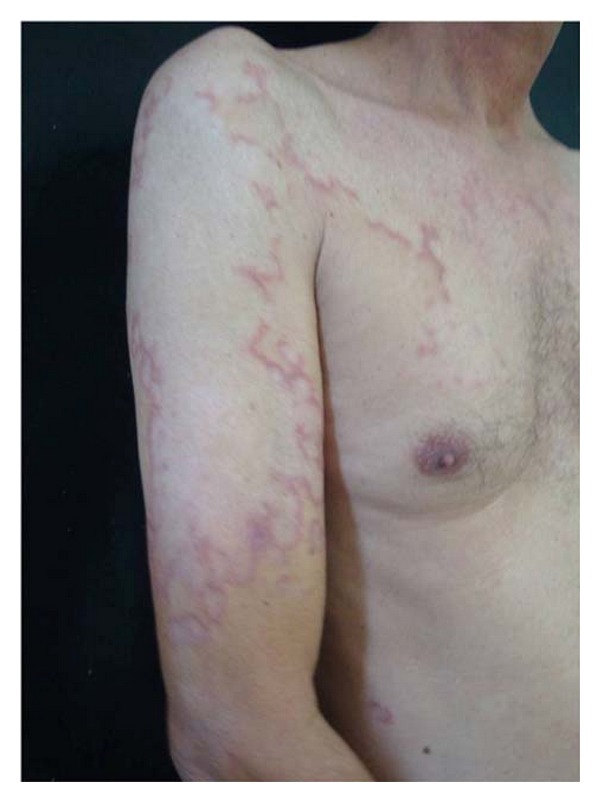
Detail of the erythematous annular lesion.

A 54-year-old man presented with a one-year complaint of asymptomatic and diffuse skin lesions increasing gradually in number and size. These lesions first appeared on his upper limbs. His occupation was a house builder and he didnot report to work without shirt. The man's personal history included diabetes. According to the patient, the control of diabetes did not change the evolution of the skin lesions. There was no known family history of similar skin changes. Dermatological examination revealed asymmetric erythematous papules and atrophic plaques with slightly elevated border and annular configuration on the neck, trunk (Figure 1), arms, and forearms (Figure 2). There was no mucosal lesion or nails change. The initial differential diagnoses included GA and leprosy. Direct mycological examination and anti-HIVwere negative.

**Figure 3:**
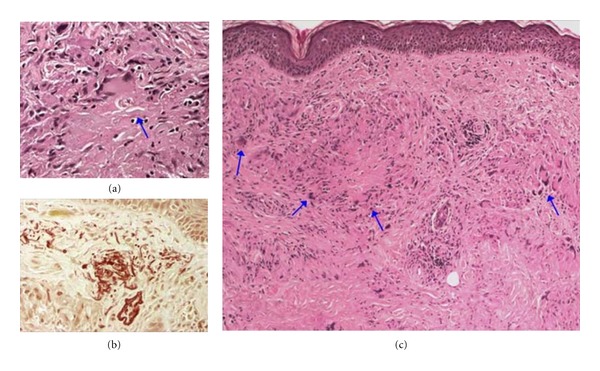
(a) Fragmentation of elastic fibers in the giant cell; H&E, 400x. (b) superficial dermis; orcein, 200x. (c) Granuloma centered by necrobiosis and multinucleate giant cells with palisading; H&E, 100x.

On histological examination of a skin biopsy taken from one of the forearm lesions, there was fragmentation of elastic fibers in the giant cell (Figure 3(a)) and superficial dermis (Figure 3(b)) and granuloma centered by necrobiosis and multinucleate giant cells with apparent palisading seen (Figure 3(c)). 

The patient was treated with topical steroids and systemic steroids for 6 months without a satisfactory response. After an ophthalmologic examination, the patient was then treated with hydroxychloroquine 400 mg/d over a period of 4 months with good response. Unfortunately, at his followup, ophthalmology examination showed macular defects and the treatment was stopped. Recurrence of the lesions was observed 3 months later.

## 3. Discussion

In 1979, Hanke et al. used for the first time the nomenclature AEGCG, when described annular skin lesions associated with granulomatous elastolytic pattern [3]. AEGCG is usually described in sun-exposed areas, such as face and neck [5], and is rarely seen on the trunk, on the back and on the extremities [1, 6]. Our patient had generalized erythematous annular lesions in the upper limbs and trunk, showing an unusual location of the lesions. Clinically, diffuse GA can represent a potential differential diagnosis, but the annular configuration may be absent giving place to a diffuse erythema and widespread papular skin lesions [7].

Since the pathogenesis of AEGCG is not entirely understood, there is a possibility that cellular immunological reactions induced by modified function of elastic fibers' antigenicity plays a role in the mechanism of AEGCG formation [8]. Such reactions would be triggered by ultraviolet radiation [8] which is important to emphasize the possibility of AEGCG being associated with systemic disorders [5]. On the other hand, GA main pathogenesis is based on a predisposition to respond to altered endogenous collagen [9].

An endocrine disease, such as diabetes mellitus (DM), could contribute for the coexistence of both lesions, and this possibility must be included in the medical investigation [9]. A recent Japanese report demonstrated the possible role of DM in the structural damage of the elastic fibers. This study indicated that 37% of Japanese patients with AEGCG who were evaluated for this metabolic disease were found to have definitive or latent DM [10].

Worthy of note is the divergence regarding the definition of AEGCG and its difference from GA. Some authors believe that AEGCG is a subclassification of GA, when the last one is in sun-exposed areas [11]. However, recent studies highlight the role of the histopathology in the distinction between the two lesions, since the presence of elastolysis and elastophagocytosis and granulomatous inflammation indicates AEGCG, in the absence of mucin deposition and necrobiosis [12]. The pattern observed in the histopathology of our patient demonstrates the typical change, described above corresponding to AEGCG, but also shows characteristic features of granuloma annulare such as palisading granulomas and necrobiosis [12]. This hybrid pattern has been described in 5 cases [9, 13–15], indicating an aetiopathogenic overlap with the possible influence of systemic diseases such as DM [9].

The existence of various treatments for AEGCG unfortunately does not guarantee satisfactory results [4]. Therapy options include clofazimine, chloroquine, and systemic corticosteroids with different success rates [1]. Our case illustrates the nonresponsiveness to corticosteroids, as well as the hydroxychloroquine demonstrating the importance of laboratory monitoring during its use in order to identify blood disorders, liver injury, and ophthalmic toxicity, the last one identified in the case.

Thus, we suggest that the mixed pattern found on histopathology could be influenced by DM and maybe other systemic diseases might play a role in the coexistence of AEGCG and GA.
